# Changes in Diagnoses and Site of Care for Patients Receiving Hospice Care From Agencies Acquired by Private Equity Firms and Publicly Traded Companies

**DOI:** 10.1001/jamanetworkopen.2023.34582

**Published:** 2023-09-25

**Authors:** Robert Tyler Braun, Mark A. Unruh, David G. Stevenson, Holly G. Prigerson, Rahul Fernandez, Leah Z. Yao, Lawrence P. Casalino

**Affiliations:** 1 Division of Health Policy and Economics, Weill Cornell Medical College, New York, New York; 2 Department of Health Policy, Vanderbilt University Medical Center, Nashville, Tennessee; 3 Center for Research on End-of-Life Care, Department of Medicine, Weill Cornell Medical College, New York, New York

## Abstract

**Importance:**

Private equity firms and publicly traded companies have been acquiring US hospice agencies; an estimated 16% of US hospice agencies are owned by private equity (PE) firms or publicly traded companies (PTC).

**Objective:**

To examine the association of PE and PTC acquisitions of hospices with Medicare patients’ site of care and clinical diagnoses.

**Design, Setting, and Participants:**

This cohort study of US hospice agencies used a novel national database of acquisitions merged with the Medicare Post–Acute Care and Hospice Public Use File for 2013 to 2020. Changes in sites of care and patient characteristics for hospice agencies acquired by PE or PTCs were compared with changes for patients in nonacquired for-profit hospice agencies.

**Exposure:**

Private equity and publicly traded company acquisitions.

**Main Outcomes and Measures:**

This study used a difference-in-differences approach within an event-study framework to examine the association of PE and PTC acquisitions of hospice agencies with changes in patient diagnoses and sites of care. Dependent variables were annual hospice-level measures of the Hierarchical Condition Category (HCC) score and proportion of patients diagnosed with cancer or dementia. Sites of care included the proportion of patients receiving hospice care in their personal home, nursing home, or assisted living facility.

**Results:**

A total of 158 hospice agencies acquired by PEs, 250 acquired by PTCs, and 1559 other for-profit hospice agencies were included. Preacquisition, hospice agencies that would later be acquired by PE or PTC served a mean (IQR) 30.1% (12.0%-44.0%) and 29.4% (13.0%-43.0%) of their patients in nursing homes respectively, a greater proportion compared with the 27.1% (8.0%-43.8%) served by for-profit hospices that were never acquired. Agencies acquired by PE between 2014 and 2019 saw a significant relative increase of 5.98% in dementia patients (1.38 percentage points; 95% CI, 0.35-2.40 percentage points; *P* = .008). In PTC-owned hospices, the proportion of patients receiving care at home increased by 5.26% (2.98 percentage points; 95% CI, 1.46-4.51 percentage points; *P* < .001), the proportion of dementia patients rose by 13.49% (3.11 percentage points; 95% CI, 2.14-4.09 percentage points; *P* < .001), and the HCC score decreased by 1.37% (−3.19 percentage points; 95% CI, −5.92 to −0.47 percentage points; *P* = .02).

**Conclusions and Relevance:**

These findings suggest that PE and PTCs select patients and sites of care to maximize profits.

## Key Points

**Question:**

Is the acquisition of hospice agencies by private equity firms and publicly traded companies associated with changes in site of care and clinical diagnoses of Medicare patients?

**Findings:**

This cohort study used a difference-in-difference analysis to examine 1967 US hospice agencies, of which 158 were acquired by private equity firms and 250 were acquired by publicly traded companies. Prior to acquisition, private equity firms and publicly traded companies targeted hospices with more patients in nursing homes; after acquisition, both agencies of private equity firms and publicly traded companies saw a rise in Medicare patients with dementia and patients who received hospice services at home, and hospices acquired by publicly traded companies were associated with lower patient Hierarchical Condition Category score after acquisition.

**Meaning:**

These results suggest that after private equity firm and publicly traded company acquisitions, hospice agencies, as compared with for-profit hospices that were never acquired, saw an increase in Medicare patients receiving care at home and those diagnosed with dementia.

## Teaser

This cohort study of US hospice agencies compares characteristics of patients, clinical diagnoses, and site of care for agencies acquired by private equity firms or publicly owned companies.

## Introduction

Over the past 3 decades, the hospice industry has evolved from relatively small, nonprofit, grassroots agencies centered on providing home-based end-of-life care for patients with cancer to one now dominated by for-profit hospices. For-profit hospices comprise nearly two-thirds of all agencies and serve a broader population living in both community and institutional settings. An understudied, emerging phenomenon is the increasing ownership of hospices by private equity (PE) firms and publicly traded companies (PTC).

In 2019, 16% of Medicare hospice patients received care from PE- or PTC-owned agencies.^1^ PE firms are for-profit entities that invest in many industries using capital raised from sources such as pension funds, sovereign wealth funds, high–net worth individuals, and university endowments. Investments by PE firms are typically private and not accessible to the general public and are not publicly traded. PE firms generally expect average annual returns of 20% or more.^2^ Much like PE investment, PTCs are for-profit entities, but with ownership distributed among public shareholders. PE and PTCs invest in the hospice sector through a “platform and roll-up” approach. Typically, the PE firm or PTC purchases a sizable, well-managed, reputable agency in the community (the platform agency). Through the platform agency, the PE firm then acquires smaller agencies (the roll-up) to increase market share, capture referrals, and create economies of scale.^3^

Hospices are appealing to PE and PTCs due to the stable Medicare payments, relatively easy market entry, and minimal capital requirements. Proponents claim that these institutional investments create economies of scale through clinical standardization, quality improvement, and integrated systems, thereby enhancing care and profitability while reducing clinicians’ administrative burdens.^4,5^ Critics argue that PE and PTCs could prioritize short-term, above-market returns, which may lead to agencies selectively enrolling and targeting patients who require less complex care and longer hospice stays, such as those with dementia and nursing home residents.^6,7,8,9,10^

In this study, we update estimates from a prior study and document the evolution of PE and PTC acquisitions of hospice agencies during the period from 2010 to 2021, including the number of deals and agencies acquired, the percentage of Medicare fee-for-service patients that received care from PE- and PTC-owned agencies, and their geographical distribution. Additionally, using a difference-in-differences approach within an event study framework, we used novel databases of PE and PTC acquisitions linked to the Medicare Post–Acute Care and Hospice Public Use File (PAC PUF) for 2013 to 2020 to examine changes in hospice patient characteristics and the sites where they received care.

## Methods

### Data

Using the Irving Levin Associates Health Care Mergers data set with previously developed methods, we calculated the number of deals involving acquisitions of freestanding hospice agencies by PE firms and PTCs from 2010 to 2021.^1,11^ This information was merged with Centers for Medicare & Medicaid Services (CMS) hospice Provider of Services files and matched on hospice agency name and address. Using CMS Certification Numbers from the Provider of Services file, we merged this information with the 2013 to 2020 PAC PUF, the most recent years available, to obtain patient and hospice characteristics.

The study was approved by the institutional review board of Weill Cornell Medical College. Informed consent was waived because publicly available databases with deidentified data were used. The study followed the Strengthening the Reporting of Observational Studies in Epidemiology (STROBE) reporting guidelines.

### Study Samples

Our study consisted of 2 samples. The first sample included all hospice agencies that were acquired by PE or PTC from 2010 to 2021. This sample was used to examine the prevalence and geographic distribution of PE- and PTC-owned hospice agencies. The second sample was limited to years 2013 to 2020 and was used for adjusted estimates based on a difference-in-differences approach within an event-study framework that compared changes in the sites of care and patient clinical characteristics after PE or PTC acquisition to concurrent changes in for-profit hospice agencies that were never acquired. The sample included hospices that were acquired between 2014 and 2019 as the treatment group (referred to as PE- or PTC-owned) and a control group (nonacquired hospices) consisting of for-profit agencies that were never acquired during the sample period. Using this sample, we were able to compare dependent variables at least 1 year before acquisition and 1 to 6 years after acquisition.

The second sample for the difference-in-differences approach within an event-study framework included only hospices present in the year in which an acquisition occurred and the year before and after the acquisition occurred. This allowed us to reduce the possibility of results being driven by compositional changes due to hospices entering or exiting the sample. We excluded nonprofit (unless a PE firm or PTC acquired a nonprofit hospice), government, and hospital-based hospices as they are fundamentally different from agencies acquired by PE and PTCs, as well as nonacquired for-profit agencies. If a hospice was acquired more than once by PE or PTC during our study period, we considered only the first investment. Hospices that were acquired prior to 2014 were excluded. We included agencies that had nonmissing observations across all primary measures to compare the same number agencies across all models (eFigure 1 in Supplement 1).

### Study Variables

#### Patient Clinical Characteristics and Sites of Care

For our primary analyses, patient characteristics examined included hospice-level Hierarchical Condition Category (HCC) score and the proportion of patients diagnosed with cancer or dementia. Sites of care included the proportion of patients receiving hospice care in their personal home, nursing home, or assisted living facility. The HCC score is a risk adjustment model used in the Medicare population to predict health care costs based on the severity of disease and demographic characteristics. Examining changes in HCC scores as well as the clinical diagnoses of dementia and cancer is crucial to understanding how financial incentive structures influence the behavior of hospices. Patients with lower HCC scores, with dementia, and/or who are living in nursing homes or assisted living facilities often have longer-term but lower intensity care needs, which can generate more profit due to the long duration of their stay in hospice and the per-diem payment structure. Conversely, patients with cancer, who typically have shorter hospice stays but more intensive needs, may be less profitable.

#### Hospice Agency Characteristics and Patient Demographics

Hospice agency characteristics included the hospice’s years in business, indicating the length of its operational existence, and the number of beneficiaries. Patient demographics include the mean age of Medicare patients and the percentage of patients dually eligible for both Medicare and Medicaid. Additionally, we also included the percentage of female patients and the percentages of White patients and patients from minoritized racial and ethnic groups.

### Statistical Analysis

We conducted 4 analyses. In our first sample, which did not exclude acquisitions prior to 2014 and after 2019, we examined the prevalence and geographic distribution of PE and PTC acquisitions from 2010 to 2021.

Second, using the second sample of hospice agencies that received their first or only acquisition between 2014 and 2019 as our treatment group, we compared agency and patient characteristics and dependent variables for PE and PTC hospices and nonacquired hospices in 2013 (preacquisition) and 2020 (postacquisition). Student *t* tests were used for comparisons of continuous variables.

Third, using our second sample, we used a difference-in-differences model within an event-study framework that addresses the differential timing of PE and PTC acquisitions to estimate changes in patient characteristics and sites of care.^12,13^ This approach compares changes in dependent variables for hospices that were acquired between 2014 and 2019 with concurrent changes in nonacquired hospices across the years 2013 to 2020. The nonacquired hospice dependent variables are assumed to represent the counterfactual outcome had acquired hospices never experienced acquisition. The event-study models allowed us to visually inspect how the treatment effect changes over time, and particularly whether there were any pretrends or anticipation effects before the treatment started. Event-study plot estimates are displayed for a 4-year time frame both prior to and after the acquisition. The aim of this approach was to mitigate the influence of potential outliers, particularly in estimates that are distant from the period of acquisition.

The difference-in-differences and event-study models included hospice agency and calendar-year fixed effects. Relative differences were derived by dividing the adjusted estimates for each outcome measure by the unadjusted mean of primary measures in the preacquisition period (year 2013). Standard errors were adjusted for clustering at the hospice agency level. The unit of analysis was the hospice agency-year. Dependent variables were Winsorized at the top and bottom 1%.

Fourth, we assessed the sensitivity of our results by including the model state fixed effects, hospice years in business, and patient demographics (average age, percentage female, percentage of dually eligible patients, and percentage of patients from minoritized racial and ethnic groups). We also conducted an analysis that includes agencies with missing data across all outcomes. This examination helped us assess whether the control group in our main results were a representative counterfactual.

Data analyses were conducted from October 7, 2022, to August 9, 2023, using Stata, release 16.0 (StataCorp LLC). Statistical tests were 2-tailed and *P* values <.05 were considered significant.

## Results

### PE and PTC Acquisition in Hospices

**Figure 1.**
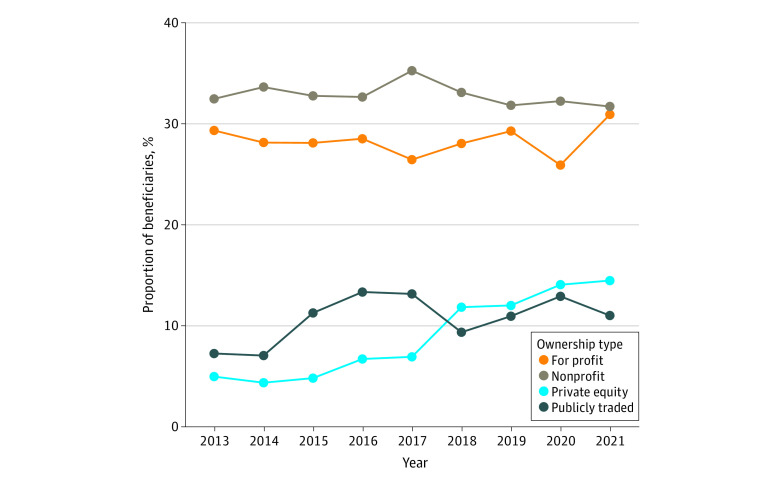
Percentage of Medicare Hospice Patients by Ownership Type Government owned hospice agencies were excluded. Data sources included were Hospice Public Use Files (Centers for Medicare & Medicaid Services [CMS]; 2013 to 2020), Providers of Service Files (CMS; 2013 to 2020), Hospice Provider Data (CMS, 2021), and Irvin Levin Associates (2010 to 2021).

Between 2010 and 2021, there were 178 PE acquisitions involving 853 agencies and 15 PTC deals with 421 agencies (eFigure 2 in Supplement 1), for a total of 193 deals and 1274 acquired hospices in the US. In 2021, 144 444 (14.4%) and 109 599 (11.0%) Medicare hospice patients received care from PE and PTC-owned hospices, respectively (Figure 1).

PE-owned hospice agencies were in 37 states, with 248 (47.5%) in the South, 129 (24.7%) in the Midwest, 129 (24.3%) in the West, and 18 (3.5%) in the Northeast. PTC-owned hospice agencies were in 40 states, with 212 (53.9%) of them in the South, 79 (20.1%) in the Midwest, 74 (18.8%) in the West, and 28 (7.1%) in the Northeast.

**Figure 2.**
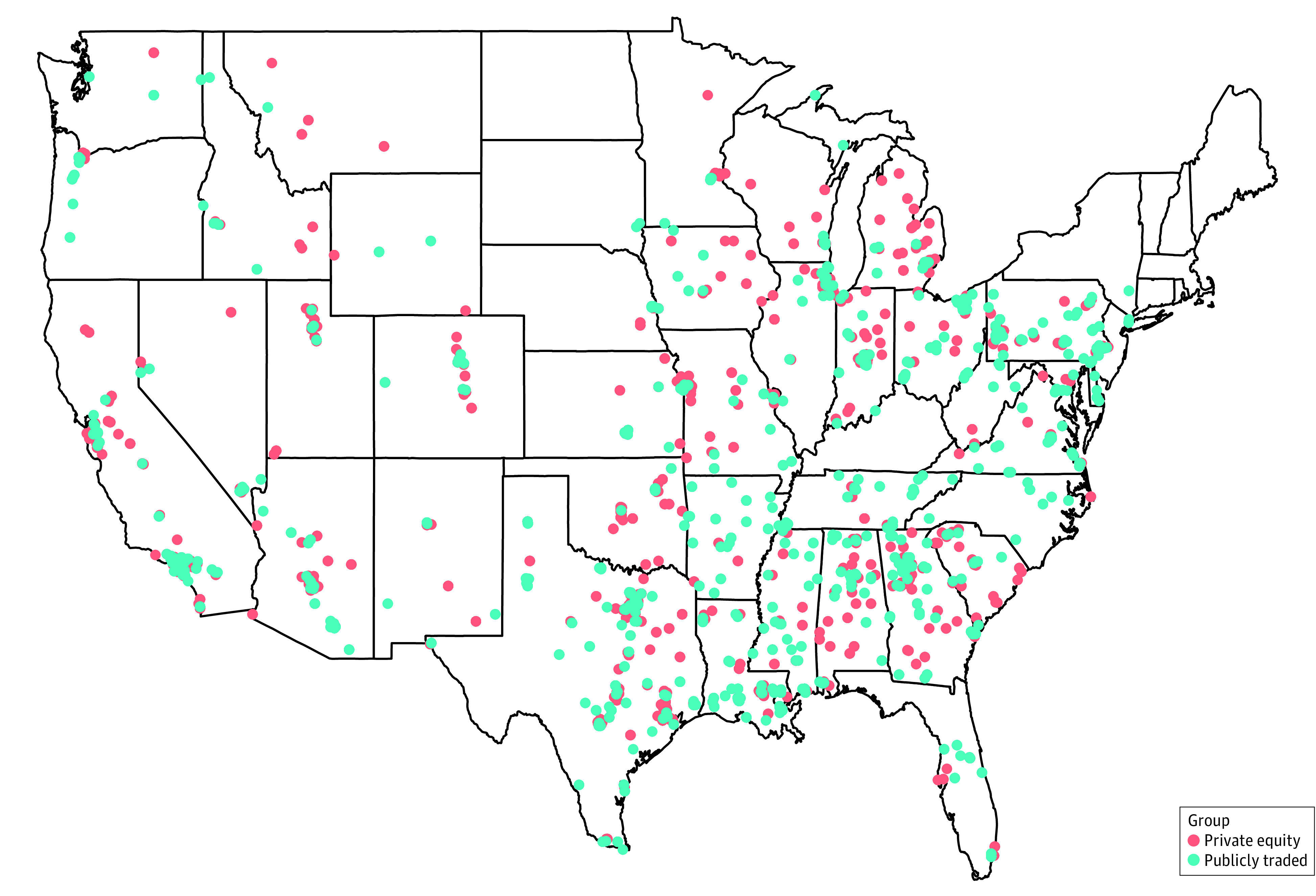
Geographical Distribution of US Hospices Owned by Institutional Investors, 2021 Data sources included were Hospice Public Use Files (Centers for Medicare & Medicaid Services [CMS]; 2013 to 2020), Providers of Service Files (CMS; 2013 to 2020), and Irvin Levin Associates (2010 to 2021).

Texas, California, and Georgia had the most acquisitions by both PE and PTCs, accounting for 108 (16.8%), 57 (9.5%), 54 (7.8%) of PE and 58 (11.5%), 34 (7.7%), and 32 (7.4%) PTC acquisitions, respectively. Michigan (27 [4.6%]) and Arizona (25 [4.2%]) also saw significant PE acquisitions, while Pennsylvania (28 [6.9%]) and Louisiana (25 [5.6%]) were prominent for PTC acquisitions (Figure 2).

### Characteristics of Patients and Hospices

Our difference-in-differences sample included 1559 for-profit agencies never acquired by PE or PTCs and 158 PE and 250 PTC hospices acquired between 2014 and 2019. Preacquisition characteristics were generally similar across groups (Table 1).

**Table 1.** Unadjusted Changes in Hospice Agency and Patient Characteristics Associated With Acquisitions by Private Equity (PE) and Publicly Traded Companies (PTC)^a^

Characteristics	Pooled sample (2013-2020), mean (IQR)	2013 (preacquisition), mean (IQR)			2020 (postacquisition), mean (IQR)			Unadjusted difference			
		For-profit	PE	PTC	For-profit	PE	PTC	PE (95% CI)	*P* value	PTC (95% CI)	*P* value
Agency characteristics											
Age of hospice, y	15.7 (9.0-21.0)	17.5 (12.0-22.0)	17.6 (13.0-23.0)	18.9 (13.0-24.0)	13.1 (7.0-17.0)	16.2 (11.0-22.5)	18.3 (13.0-24.0)	−3.10 (−5.16 to −1.05)	.003	−3.76 (−5.47 to −2.05)	<.001
Beneficiaries, No.	361.7 (147.0-432.0)	337.8 (138.0-384.0)	392.4 (191.0-472.5)	437.5 (193.0-597.0)	371.5 (151.0-415.0)	585.1 (261.0-834.0)	408.5 (212.0-464.8)	−159.07 (−273.00 to −45.08)	.006	63.01 (−28.01 to 154.03)	.18
Patient characteristics											
Age, y	82.3 (81.0- 84.0)	82.2 (81.0-84.0)	82.1 (80.5-83.0)	81.8 (80.0-83.0)	82.3 (81.0-84.0)	81.8 (80.0-83.0)	81.7 (80.0-83.0)	0.38 (−0.21 to 0.96)	.20	0.19 (−0.29 to 0.66)	.46
Dual eligible, %	29.6 (20.0-38.0)	31.7 (21.0-41.0)	29.9 (22.0-37.0)	30.1 (21.0-39.0)	28.3 (19.0-37.0)	28.2 (20.0-36.0)	26.5 (20.0-33.0)	−1.74 (−5.20 to 1.69)	.32	−0.04 (−2.80 to 2.69)	.23
Female, %	59.6 (56.0-63.0)	60.6 (57.0-64.0)	60.7 (57.5-64.0)	60.1 (57.0-63.0)	59.0 (56.0-62.0)	58.1 (55.0-61.0)	58.4 (55.0-61.0)	−0.98 (−0.34 to 2.32)	.15	0.26 (−0.77 to 1.30)	.61
White, %	80.2 (71.0-92.0)	80.9 (73.0-93.0)	84.6 (77.5-94.0)	82.1 (73.0-94.0)	77.9 (68.0-81.0)	83.9 (76.0-94.0)	80.6 (71.0-92.0)	−2.30 (−6.10 to 1.49)	.88	−1.63 (−4.77 to 1.51)	.31
Minority racial or ethnic group, %	16.4 (2.0-26.0)	15.3 (0-24.0)	12.2 (0-20.0)	16.0 (3.0-26.0)	18.1 (4.0-28.0)	12.9 (3.0-20.0)	15.8 (4.0-25.0)	2.15 (−1.87 to 6.18)	.09	2.80 (−0.59 to 6.20)	.07
Place of care											
Home, %	56.0 (38.0-74.0)	57.8 (37.0-79.8)	54.5 (36.8-70.3)	53.7 (37.0-79.0)	56.8 (48.0-76.0)	50.1 (37.0-64.0)	55.4 (42.0-72.0)	3.37 (−2.97 to 9.72)	.29	−2.64 (−7.84 to 2.56)	.32
Assisted living, %	18.4 (4.0-27.0)	12.3 (1.0-20.0)	14.0 (3.8-21.5)	14.0 (4.0-20.0)	22.4 (6.0-33.0)	22.9 (10.0-32.8)	21.7 (7.0-32.5)	1.23 (−3.53 to 5.99)	.61	2.50 (−1.49 to 6.50)	.22
Nursing home, %	30.6 (8.0-43.0)	27.1 (8.0-43.8)	30.1 (12.0-44.0)	29.4 (13.0-43.0)	33.1 (6.0-45.3)	38.7 (17.3-48.0)	39.5 (14.0-46.5)	−2.70 (−10.56 to 5.17)	.50	−4.02 (−10.5 to 2.46)	.22
Clinical characteristics											
Dementia, %	23.7 (17.0-30.0)	23.8 (17.0-30.0)	21.1 (15.0-26.0)	21.0 (17.0-25.0)	23.8 (17.0-30.0)	22.9 (17.0-28.0)	23.7 (17.0-29.0)	−1.96 (−4.34 to 0.40)	.10	−2.60 (−4.45 to −0.71)	.007
Cancer, %	22.3 (16.0-27.0)	24.1 (17.3-30.0)	23.1 (18.0-28.3)	23.7 (19.0-28.0)	20.0 (15.0-25.0)	19.5 (15.0-23.0)	20.4 (16.0-24.5)	−0.43 (−2.47 to 1.61)	.67	−0.76 (−2.43 to 0.91)	.37
Risk score	2.45 (2.20-2.60)	2.34 (2.20-2.50)	2.25 (2.10-2.40)	2.34 (2.20-2.50)	2.54 (2.30-2.70)	2.44 (2.30 to 2.60)	2.47 (2.30-2.60)	0.02 (−0.06 to 0.09)	.68	0.06 (0.01 to 0.12)	.03

^a^ The sample includes 158 freestanding hospices owned by PE and 250 owned by PTC acquired between 2014 and 2019. In addition, there are 1559 for-profit hospices that were never acquired by a PE firm or PTC in the comparison group. We only included hospices without missing observations across all outcomes. Outcomes were Winsorized at the top and bottom 1%.

The age of hospices was similar between PE (mean [IQR] age, 17.6 [13.0-23.0] years), PTC (18.9 [13.0-24.0] years), and other for-profit hospices (17.5 [12.0-22.0] years) in the preacquisition period. Hospice agencies that were acquired by PE and PTC were typically larger (mean [IQR] beneficiaries: PE, 392.4 [191.0-472.5]; PTC, 437.5 [193.0-597.0]) relative to other for-profit hospices (337.8 [138.0-384.0]). There was little variation in patient characteristics postacquisition, including age, dual eligibility, gender, and race. Before acquisition, hospice agencies that were acquired by PE firms or PTCs served a mean (IQR) 30.1% (12.0%-44.0%) and 29.4% (13.0%-43.0%) of their patients in nursing homes. This is in contrast with for-profit hospices, which served a lower percentage (27.1% [8.0%-43.8%]) of their patients in the same setting. There were no significant differences in patient clinical characteristics prior to acquisition.

### Changes in Patient Clinical Characteristics and Sites of Care

Post–PE acquisition, there was a significant 5.98% (1.38 percentage points, 95% CI, 0.35 to 2.40 percentage points; *P* = .008) relative increase in patients with dementia. Post–PTC acquisition, there was a 5.26% relative increase (2.98 percentage points; 95% CI, 1.46 to 4.51 percentage points; *P* < .001) in the proportion of patients receiving care at home, a 13.49% relative increase in patients with dementia (3.11 percentage points; 95% CI, 2.14-4.09 percentage points; *P* < .001), and a 1.37% decrease (−3.19 percentage points; 95% CI, −5.92 to −0.47 percentage points; *P* = .02) in the HCC risk score (Table 2).

**Table 2.** Adjusted Changes in Outcomes Associated With Acquisitions of Hospice Agencies by Private Equity (PE) Firms and Publicly Traded Companies (PTC)^a^

Outcome	Pooled sample means (2013)	Postacquisition					
		PE adjusted difference-in-difference^b^	Relative change, %	*P* value	PTC adjusted difference-in-difference^b^	Relative change, %	*P* value
Place of care							
Home, %	56.69	1.91 (0.16 to 3.67)	3.37	.03	2.98 (1.46 to 4.51)	5.26	<.001
Assisted living, %	12.81	−2.06 (−3.73 to −0.38)	−16.08	.02	−1.19 (−2.51 to 0.14)	−9.29	.08
Nursing home, %	27.84	−1.36 (−4.3 to 1.57)	−4.89	.36	0.25 (−2.24 to 2.75)	0.91	.84
Patient characteristics							
Dementia, %	23.06	1.38 (0.35 to 2.4)	5.98	.008	3.11 (2.14 to 4.09)	13.49	<.001
Cancer, %	23.9	0.17 (−0.63 to 0.97)	0.71	.67	−0.45 (−1.12 to 0.22)	−1.88	.19
Risk score	2.33	1.19 (−1.89 to 4.27)	0.51	.45	−3.19 (−5.92 to −0.47)	−1.37	.02

^a^ The sample includes 158 freestanding hospices owned by PE and 250 owned by PTC acquired between 2014 and 2019. In addition, there are 1559 for-profit hospices that were never acquired by a private equity or publicly traded company in the comparison group. We only included hospices without missing observations across all outcomes. Outcomes were Winsorized at the top and bottom 1%.

^b^ Callaway and Sant’Anna^13^ difference-in-differences method was used. Models included fixed effects for agency and year. The unit of analysis was the hospice agency-year; standard errors were adjusted for clustering at the level of the hospice agency.

**Figure 3.**
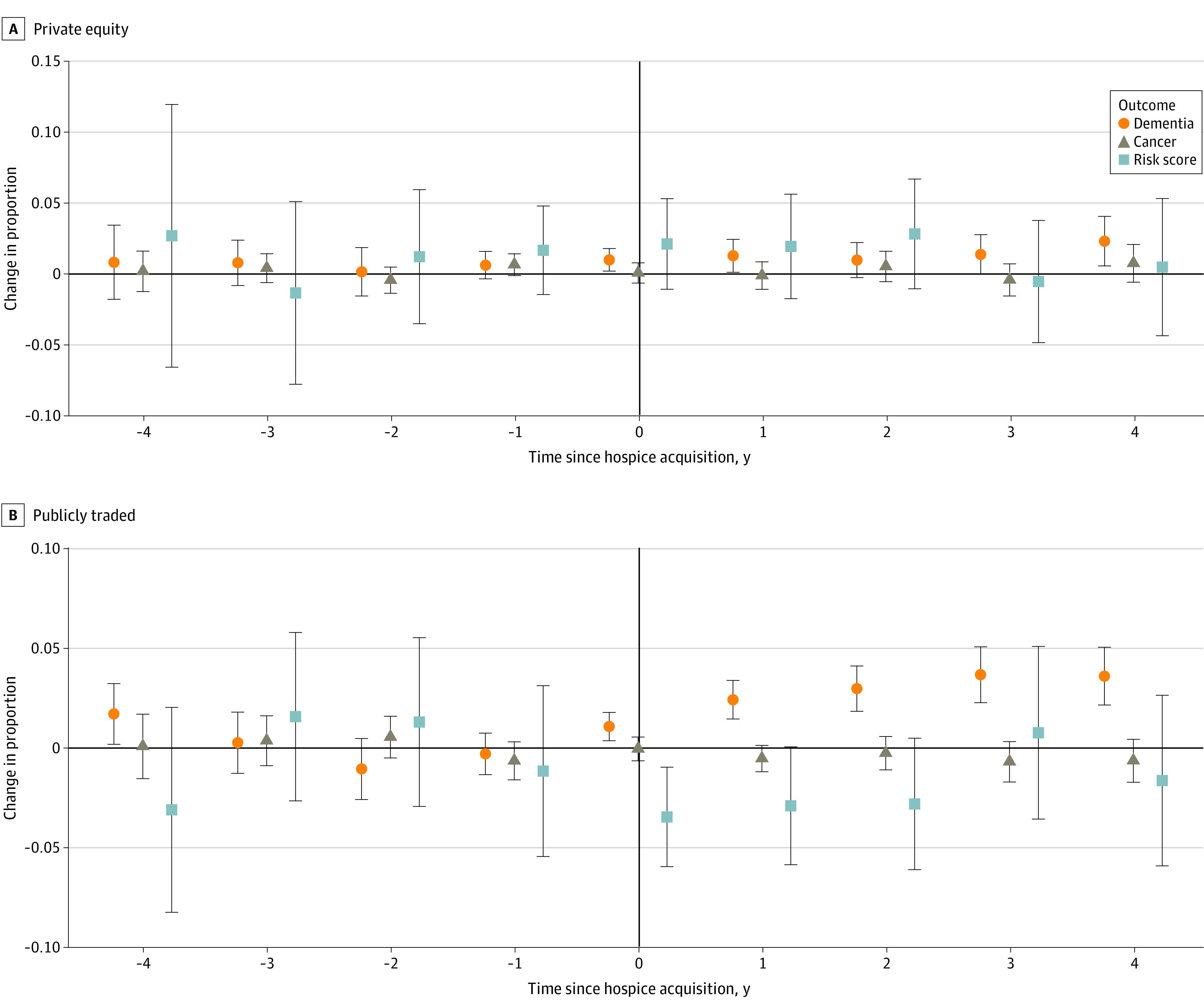
Adjusted Differences in Outcomes Between Treatment and Comparison Agencies Relative to the Year of Acquisition by Private Equity or a Publicly Traded Company The sample included 158 freestanding hospices owned by private equity and 250 owned by publicly traded companies, which were acquired between 2014 and 2019. In addition, there are 1559 for-profit hospices in the comparison group. We only included hospices without missing observations across all outcomes. Outcomes were Winsorized at the top and bottom 1%. Callaway and Sant’Anna^13^ difference-in-differences method was used. Models included fixed effects for agency and year. The unit of analysis was the hospice agency-year; standard errors were adjusted for clustering at the level of the hospice agency.

Adjusted event study plots showed a small increase in dementia patients post–PE acquisition and a consistent rise post–PTC acquisition, along with a decrease in HCC risk scores (Figure 3). Changes in the proportion of patients with dementia ranged from a 5.55% relative increase (1.28 percentage points; 95% CI, 0.12 to 2.44 percentage points; *P* = .03) 1-year postacquisition to a 10.01% relative increase (2.31 percentage points; 95% CI, 0.56 to 4.06 percentage points; *P* = .01) 4-years post–PE acquisition. Changes in the proportion of patients with dementia were more dramatic post–PTC acquisition, ranging from 10.62% (2.45 percentage points; 95% CI, 1.49 to 3.40 percentage points; *P* < .001) 1-year postacquisition to 15.69% (3.62 percentage points; 95% CI, 2.18 to 5.05 percentage points; *P* < .001) 4-years postacquisition.

Hospice care provided in the home increased, while the percentage of patients in assisted living decreased following PE and PTC acquisition (eFigure 3 in Supplement 1). Care provided in the nursing home remained unchanged but remained higher for PE- and PTC-owned hospices across the study period. After adding state fixed effects (eTable 1 in Supplement 1) and including hospice agency characteristics and patient demographics (eTable 2 in Supplement 1) to models in sensitivity analyses, results were similar to our primary analyses. By removing the nonmissing observation exclusion criteria, our results remained largely similar, with the exception that the proportion of dementia patients after PE acquisition was significant at the 0.1 level (eTable 3 in Supplement 1).

## Discussion

In this national cohort study using an adjusted difference-in-differences analysis, hospice agencies acquired by PE firms and PTCs saw 5.98% and 13.49% relative increases, respectively, in patients with dementia after acquisition compared with for-profit hospice agencies that were never acquired. Hospice agencies had a 1.37% decrease in HCC risk score post–PTC acquisition. Before being acquired, hospice agencies that eventually were acquired by PE firms (30.1%) or PTCs (29.4%) were more likely to serve patients in nursing homes compared with for-profit hospice agencies (27.1%) that were never acquired. PE and PTC acquired hospice agencies saw increases of 3.37% and 5.26% in care being provided in a patient’s personal home, respectively.

To our knowledge, this is the first study to examine the impact of PE and PTC acquisitions on clinical characteristics and sites of care. Recent studies of PE acquisitions in nursing homes and in physician groups suggest that quality of care decreases and cost of care goes up following these transactions after PE acquisition.^11,14,15,16,17,18^ The shift in clinical characteristics, specifically the significant increase in dementia patients, suggests a potential change of operational strategy to focus on certain patient populations that maximize profitability following acquisitions. These changes are consistent with profit maximization strategies that seek to take advantage of a flat per-diem reimbursement structure that rewards serving patients with longer lengths of stay, lower acuity, and who are colocated in nursing homes and assisted living facilities.

Some caution should be exercised in interpreting these findings, especially as they might relate to quality of care. It is essential to investigate these transactions further to understand whether they are associated with patient outcomes or possibly driven by other factors. A more detailed analysis using hospice claims, for instance, could examine the impact of PE and PTC acquisitions on service use outcomes such as lengths of stay, skilled nursing visits, and visits in the last days of life. Additionally, to gain a more comprehensive understanding of the impact of these changes on patient experience and burdensome care, it would be beneficial to analyze additional quality measures, such as data from the Consumer Assessment of Healthcare Providers and Systems (CAHPS) Hospice Survey and other outcomes from claims-based data.^7,19^

Medicare is the primary revenue source for hospices, yet the opaque nature of hospice ownership can obscure the identification of PE and PTC acquisitions. Facilitating greater transparency in hospice ownership and operations is a first step in achieving improved accountability for care. Although the CMS has recently released hospice ownership data via the Hospice Compare website, it does not fully capture all of the acquisitions identified in our study or related organizations. For instance, it is not possible with current data to identify hospice agencies with common ownership, whether PE, PTC, or other chains. To safeguard patient care quality and accessibility in the context of the changing hospice market, it may be necessary for policymakers to require greater transparency in reporting these acquisitions and implement additional oversight measures. In fact, US lawmakers and the National Hospice and Palliative Care Organization came out in support of several such measures following a ProPublica investigation of PE and other for-profit investment in the hospice sector.^20^ These reforms could involve improved reporting of ownership details in resources intended for patients and families seeking information on hospice care quality and mandating that a hospice report within 30 to 90 days that a change of ownership took place. Patients and families are essentially health care consumers and have a right to know about aspects of hospice care that are likely to have implications for access to and quality of the care provided to terminally ill patients with sufficient auditing and enforcement mechanisms for compliance.

### Limitations

Our study has limitations. First, our results were representative of larger hospices due to the CMS redaction of information based on counts of less than 10 patients in the PAC PUF. Second, we identified hospice acquisitions using the Irving Levin Associates Health Care M&A data set, which relies on public announcements of hospice acquisitions and might not include smaller acquisitions or acquisitions that have nondisclosure agreements. In these cases, agencies acquired on behalf of a PE firm or PTC may appear to be for-profit hospices that were never acquired; this would bias our estimates toward the null. Third, we were unable to track PE firm or PTC exits from hospices for most of acquisitions, which also may bias our estimates toward no association of ownership with outcomes. Fourth, we were not able to identify the precise timing of acquisitions within a year, as the Medicare PAC PUF only provides updates annually, potentially introducing some measurement error into our analysis.

## Conclusions

This cohort study suggests that hospices owned by PE firms and PTCs may target Medicare patients likely to be more profitable, specifically those in sites of care and/or with certain clinical conditions, such as dementia, that are associated with lower complexity of care. Additionally, prior to acquisition, PE firms and PTCs appear to have targeted agencies with more patients in nursing homes.

## Notes

Supplement 1. **eFigure 1.** Sample Selection **eFigure 2.** The Number of Transactions and Agencies Acquired by Private Equity Firms and Publicly Traded Corporations, 2013-2021 **eFigure 3.** Adjusted Differences in Sites of Care Between Treatment and Control Agencies Relative to the Year of Hospice Acquisition **eTable 1.** Adjusted Differences in Sites of Care and Patient Characteristics Between Treatment and Control Agencies Relative to the Year of Hospice Acquisition, with State Fixed Effects **eTable 2.** Adjusted Differences in Sites of Care and Patient Characteristics Between Treatment and Control Agencies Relative to the Year of Hospice Acquisition, with State Fixed Effects and Controls **eTable 3.** Removal of Missingness Exclusion Criteria

Supplement 2.
Data Sharing Statement
