# Supplementary material for: Changes in Diagnoses and Site of Care for Patients Receiving Hospice Care From Agencies Acquired by Private Equity Firms and Publicly Traded Companies
Source: JAMA Netw Open. 2023 Sep 25;6(9):e2334582. doi: 10.1001/jamanetworkopen.2023.34582 (PMC10520742; doi:10.1001/jamanetworkopen.2023.34582)
Supplement: Supplement 1. — eFigure 1. Sample Selection eFigure 2. The Number of Transactions and Agencies Acquired by Private Equity Firms and Publicly Traded Corporations, 2013-2021 eFigure 3. Adjusted Differences in Sites of Care Between Treatment and Control Agencies Relative to the Year of Hospice Acquisition eTable 1. Adjusted Differences in Sites of Care and Patient Characteristics Between Treatment and Control Agencies Relative to the Year of Hospice Acquisition, with State Fixed Effects eTable 2. Adjusted Differences in Sites of Care and Patient Characteristics Between Treatment and Control Agencies Relative to the Year of Hospice Acquisition, with State Fixed Effects and Controls eTable 3. Removal of Missingness Exclusion Criteria [file jamanetwopen-e2334582-s001.pdf]

Supplemental Online Content

Braun RT, Unruh MA, Stevenson DG, et al. Changes in diagnoses and site of care for patients receiving hospice care from agencies acquired by private equity firms and publicly traded company. *JAMA Netw Open.* 2023;6(9):e2334582. doi:10.1001/jamanetworkopen.2023.34582

eFigure 1. Sample Selection

eFigure 2. The Number of Transactions and Agencies Acquired by Private Equity Firms and Publicly Traded Corporations, 2013-2021

eFigure 3. Adjusted Differences in Sites of Care between Treatment and Control Agencies Relative to the Year of Hospice Acquisition

eTable 1. Adjusted Differences in Sites of Care and Patient Characteristics between Treatment and Control Agencies Relative to the Year of Hospice Acquisition, with State Fixed Effects

eTable 2. Adjusted Differences in Sites of Care and Patient Characteristics between Treatment and Control Agencies Relative to the Year of Hospice Acquisition, with State Fixed Effects and Controls

eTable 3. Removal of Missingness Exclusion Criteria

This supplemental material has been provided by the authors to give readers additional information about their work.

© 2023 Braun RT et al. *JAMA Network Open.*

eFigure 1. Sample Selection

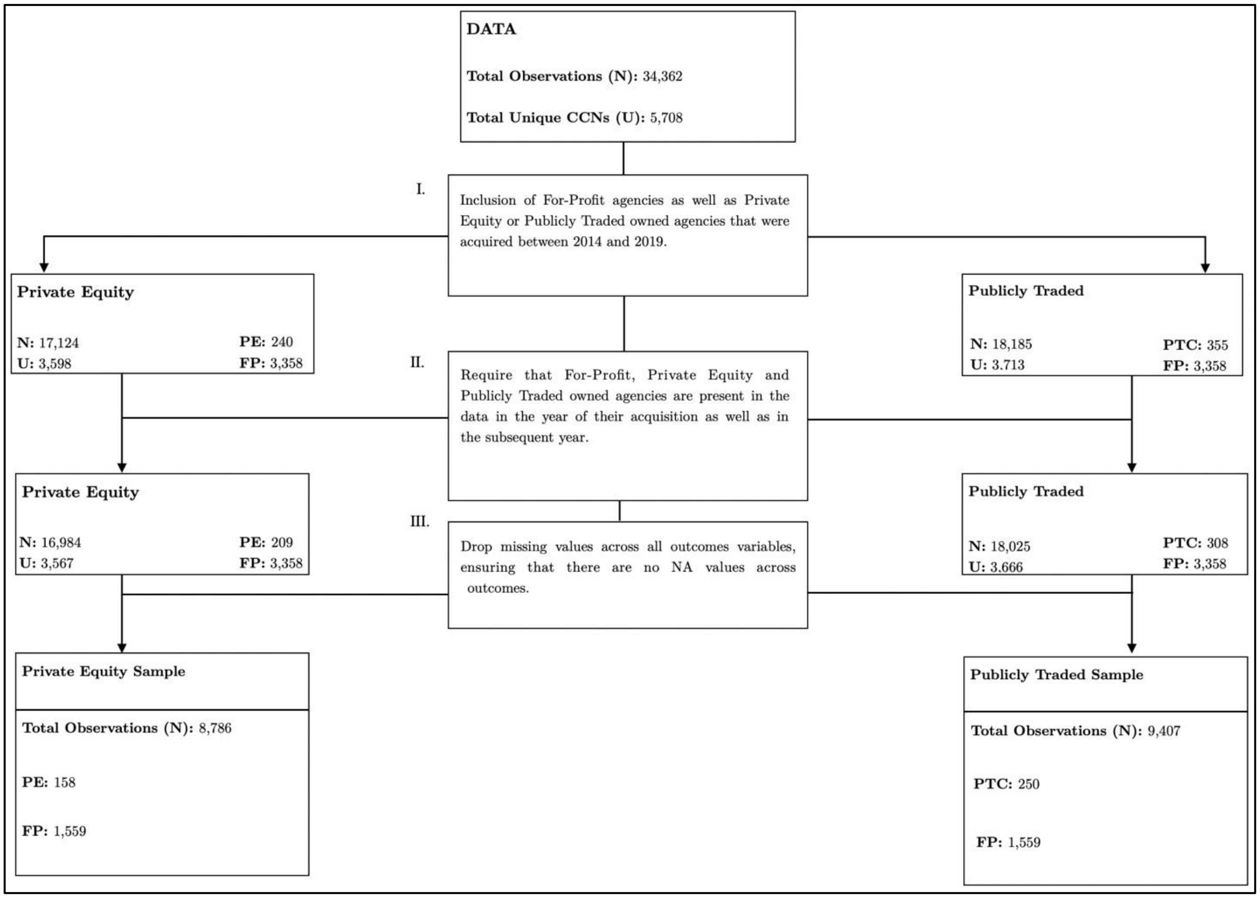

For the sample selection, we start with the base dataset consisting of variables from the CMS public use files (PUF). The dataset consists of 34,362 observations from years 2013 to 2020, with 5,708 unique CMS certification numbers (CCNs). To get to our final analytic sample, we implement the following three step process.

First, we include private equity (PE) and publicly traded company (PTC) owned facilities that have been acquired between 2014 and 2019, this brings the dataset to 17,124 and 18,185 total observations, with 3,598 and 3,713 unique CCNs, for PE and PTC samples respectively. Secondly, we implement a rule requiring for-profit (FP), PE, and PTC facilities to be present in the sample at the year of their acquisition as well as in the subsequent year, bringing the dataset to 16,984 and 18,025 total observations, with 3,567 and 3,666 unique CCNs, for PE and PTC samples respectively. Lastly, for our selected outcome variables, we implement a final criterion requiring that there be no NA/missing values present across each observation's outcomes, i.e, ensuring no NA/missing values in the entirety of the final analytic samples. This yields our final PE and PTC samples, with the FP comparison group, from 2013 to 2020.

For the final analytic PE sample, we have a total of 8,786 observations with 158 unique PE owned facilities and 1,559 unique FP facilities which make up our comparison group. The final PTC analytic sample has a total of 9,407 observations with 250 unique PTC facilities and the same FP comparison

© 2023 Braun RT et al. *JAMA Network Open.*

group with 1,559 unique facilities. The final analytic samples ensure that across all models, PE and PTC agencies are compared to the same for-profit agencies and the same agencies are compared across all outcomes.

© 2023 Braun RT et al. *JAMA Network Open.*

eFigure 2. The Number of Transactions and Agencies Acquired by Private Equity Firms and Publicly Traded Corporations, 2013-2021

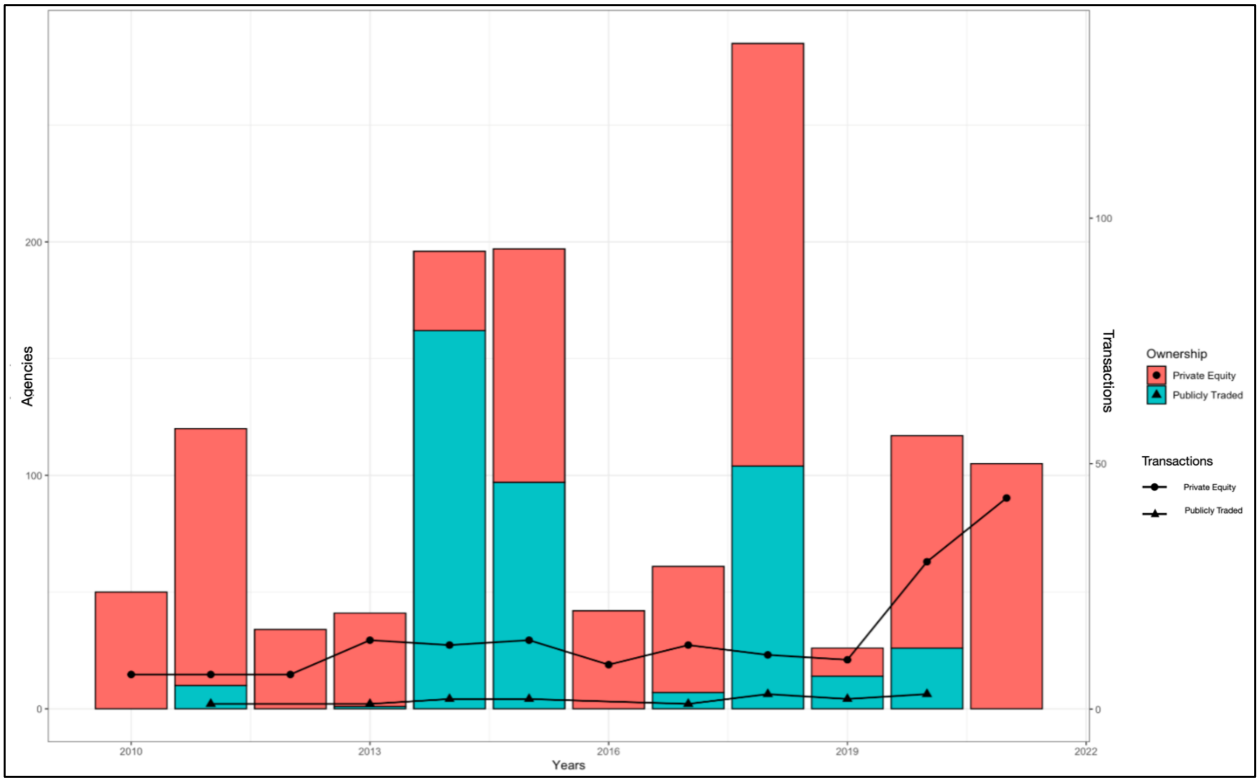

NOTE: Transactions (represented by the black lines) refer to unique acquirer acquisition events, while agencies (represented by the bars) refer to the number of hospice agencies involved in those transactions.

© 2023 Braun RT et al. *JAMA Network Open.*

eFigure 3. Adjusted Differences in Sites of Care between Treatment and Control Agencies Relative to the Year of Hospice Acquisition

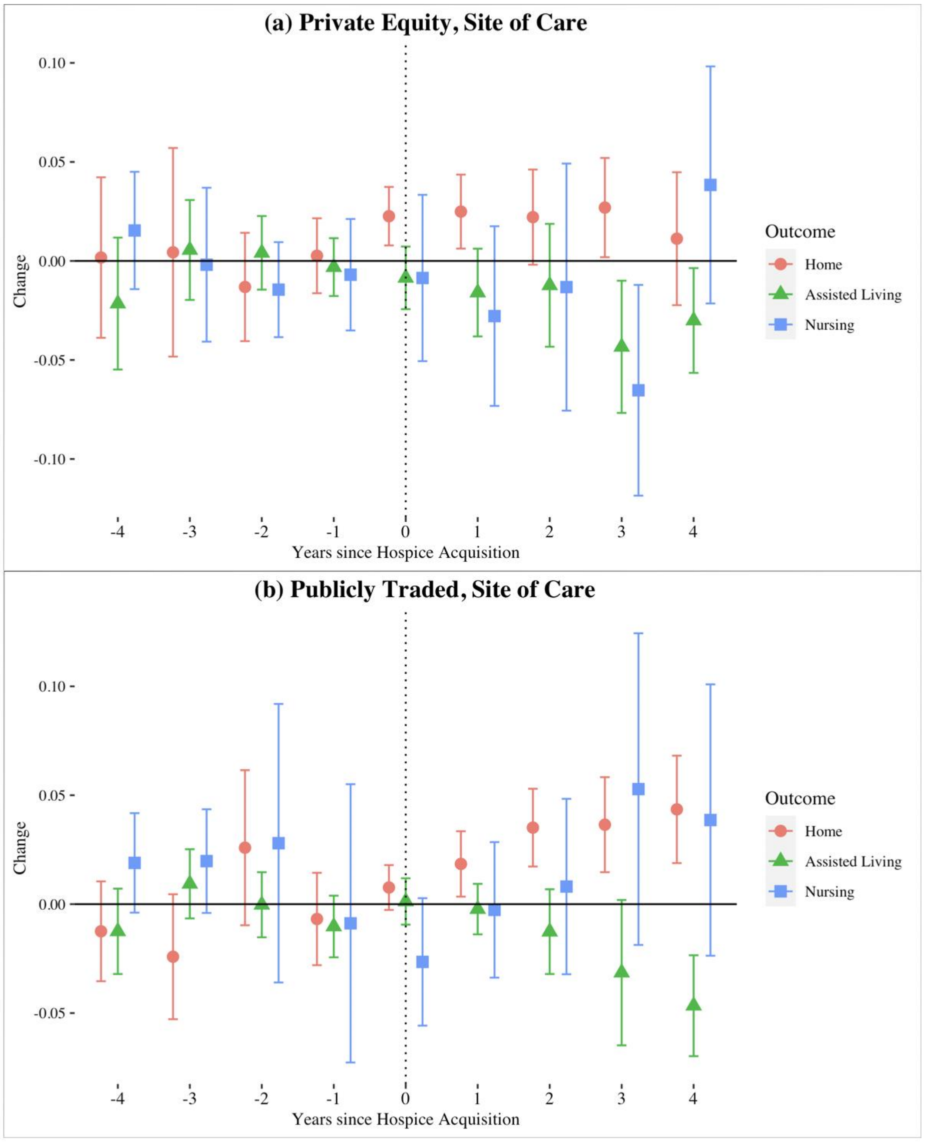

NOTES: The sample includes 158 freestanding hospices owned by PE and 250 owned by PTCs, which were acquired between 2014 and 2019. In addition, there are 1,559 for-profit hospices in the comparison group. We only included hospices without missing observations across all outcomes. Callaway and Sant'Anna (2021) difference-in-differences method was used for estimation. Models included fixed effects for agency and year. Outcomes were Winsorized at the top and bottom 1%. The unit of analysis was the hospice agency-year; standard errors were adjusted for clustering at the level of the hospice agency.

© 2023 Braun RT et al. *JAMA Network Open.*

eTable 1. Adjusted Differences in Sites of Care and Patient Characteristics between Treatment and Control Agencies Relative to the Year of Hospice Acquisition, with State Fixed Effects

		Post-Acquisition					
	**Pooled Sample Means (2013)**	**PE Adjusted Difference-in-Difference**	**Relative Change, %**	**P-Value**	**PTC Adjusted Difference-in-Difference**	**Relative Change, %**	**P-Value**
**Outcomes**							
**Place of Care**							
Home, %	56.69	1.91 [0.16, 3.7]	3.37	0.033	3 [1.46, 4.51]	5.29	0
Assisted Living, %	12.81	-2.1 [-3.7, -0.39]	-16.39	0.016	-1.2 [-2.50, 0.14]	-9.37	0.079
Nursing Home, %	27.84	-1.4 [-4.3, 1.6]	-5.03	0.362	0.25 [-2.22, 2.75]]	0.9	0.843
**Patient Characteristics, %**							
Dementia, %	23.06	1.38 [0.35, 2.4]	5.98	0.008	3.1 [2.14, 4.09]	13.44	0
Cancer, %	23.9	0.17 [-0.63, 0.97]	0.71	0.674	-0.45 [-1.12, 0.22]	-1.88	0.185
Risk Score	2.33	1.19 [-1.89, 4.27]	0.51	0.45	-3.19 [-5.92, -0.47]	-1.37	0.021

NOTES: The sample includes 158 freestanding hospices owned by PE and 250 owned by PTCs, which were acquired between 2014 and 2019. In addition, there are 1,559 for-profit hospices in the comparison group. We only included hospices without missing observations across all outcomes. Callaway and Sant'Anna (2021) difference-in-differences method was used for estimation. Models included fixed effects for agency and year. Outcomes were Winsorized at the top and bottom 1%. The unit of analysis was the hospice agency-year; standard errors were adjusted for clustering at the level of the hospice agency.

© 2023 Braun RT et al. *JAMA Network Open.*

eTable 2. Adjusted Differences in Sites of Care and Patient Characteristics between Treatment and Control Agencies Relative to the Year of Hospice Acquisition, with State Fixed Effects and Controls

		Post-Acquisition					
	**Pooled Sample Means (2013)**	**PE Adjusted Difference-in-Difference**	**Relative Change, %**	**P-Value**	**PTC Adjusted Difference-in-Difference**	**Relative Change, %**	**P-Value**
**Outcomes**							
**Site of Care**							
Home, %	55.57	1.2 [-0.68, 3.08]	2.16	0.21	2.6 [0.99, 4.21]	4.68	0.002
Assisted Living, %	13.34	-1.88 [-3.7, -0.06]	-14.09	0.042	-1.04 [-2.5, 0.41]	-7.8	0.16
Nursing Home, %	28.34	-1.39 [-4.6, 1.8]	-4.9	0.4	0.15 [-2.64, 2.95]	0.53	0.91
**Patient Characteristics, %**							
Dementia, %	22.58	1.3 [0.225, 2.38]	5.76	0.018	2.84 [1.78, 3.9]	12.58	0
Cancer, %	23.9	0.26 [-.533, 1.05]	1.09	0.52	-0.09 [-0.78, 0.60]	-0.38	0.8
Risk Score	2.33	1.04 [-2.16, 4.24]	0.45	0.53	-3.35 [-6.22, -0.48]	-1.44	0.02

NOTES: The sample includes 136 freestanding hospices owned by PE and 209 owned by PTCs, which were acquired between 2014 and 2019. In addition, there are 1,279 for-profit hospices in the comparison group. We only included hospices without missing observations across all outcomes. Callaway and Sant'Anna (2021) difference-in-differences method was used for estimation. Models included fixed effects for agency and year. Outcomes were Winsorized at the top and bottom 1%. The unit of analysis was the hospice agency-year; standard errors were adjusted for clustering at the level of the hospice agency.

© 2023 Braun RT et al. *JAMA Network Open.*

eTable 3. Removal of Missingness Exclusion Criteria

		Post Acquisition					
	**Pooled Sample Means (2013)**	**PE Adjusted Difference in Difference**	**Relative Change, %**	**P-Value**	**PTC Adjusted Difference in Difference**	**Relative Change, %**	**P-Value**
**Place of Care**							
Home, %	64	2.0 [0.00 to 4.3]	3.13	0.06	2.0 [0.1 to 3.23]	3.13	0.033
Assisted Living, %	17	-1.0 [-2.0 to 0.30]	-5.75	0.11	-0.1 [-1.2 to 1.1]	-0.58	0.831
Nursing Home, %	24	-2.0 [-4.2 to 1.0]	-8.61	0.189	1.0 [-1.5 to 2.6]	4.30	0.577
**Clinical Characteristics**							
Dementia, %	25	1.0 [-0.1 to 2.0]	4.01	0.10	3.0 [2.0 to 4.0]	12.02	<0.001
Cancer, %	23	0.5 [-0.4 to 2.0]	2.17	0.28	-0.4 [-1.0 to 0.2]	-1.74	0.209
Risk Score	2.45	1.0 [-1.5 to 4.0]	0.41	0.35	-4.0 [-6.3 to -1.4]	-1.63	0.002

NOTES: The sample includes 203 freestanding hospices owned by PE and 302 owned by PTC, which were acquired between 2014 and 2019. In addition, there are 3,358 for-profit hospices, that were never acquired by a private equity or publicly traded company, in the comparison group. We only included hospices without missing observations across all outcomes. Outcomes were Winsorized at the top and bottom 1%.
Callaway and Sant'Anna (2021) difference-in-differences method was used. Models included fixed effects for agency and year.
The unit of analysis was the hospice agency-year; standard errors were adjusted for clustering at the level of the hospice agency.

© 2023 Braun RT et al. *JAMA Network Open.*
