# Supplementary material for: Changes in Diagnoses and Site of Care for Patients Receiving Hospice Care From Agencies Acquired by Private Equity Firms and Publicly Traded Companies
Source: JAMA Netw Open. 2023 Sep 25;6(9):e2334582. doi: 10.1001/jamanetworkopen.2023.34582 (PMC10520742; doi:10.1001/jamanetworkopen.2023.34582)
Supplement: Supplement 2. — Data Sharing Statement [file jamanetwopen-e2334582-s002.pdf]

Data Sharing Statement

Braun. Changes in Diagnoses and Site of Care for Patients Receiving Hospice Care From Agencies Acquired by Private Equity Firms and Publicly Traded Companies. *JAMA Netw Open*. Published September 19, 2023. doi:10.1001/jamanetworkopen.2023.34582

Data
Data available: No

Additional Information
Explanation for why data not available: DUA does not allow it
